# Pyrolysis-induced shrinking of three-dimensional structures fabricated by two-photon polymerization: experiment and theoretical model

**DOI:** 10.1038/s41378-019-0079-9

**Published:** 2019-08-26

**Authors:** Braulio Cardenas-Benitez, Carsten Eschenbaum, Dario Mager, Jan G. Korvink, Marc J. Madou, Uli Lemmer, Israel De Leon, Sergio O. Martinez-Chapa

**Affiliations:** 1 School of Engineering and Sciences, Tecnologico de Monterrey, Av. Eugenio Garza Sada 2501 Sur, 64849 Monterrey, NL Mexico; 2 Light Technology Institute, Karlsruhe Institute of Technology (KIT), Engesserstraße 13, 76131 Karlsruhe, Germany; 3 Institute of Microstructure Technology, Karlsruhe Institute of Technology (KIT), Hermann-von-Helmholtz-Platz 1, 76344 Eggenstein-Leopoldshafen, Germany; 4 Department of Mechanical and Aerospace Engineering, University of California, Irvine, 4200 Engineering Gateway, Irvine, CA 92697 USA

**Keywords:** NEMS, Nanowires

## Abstract

The introduction of two-photon polymerization (TPP) into the area of Carbon Micro Electromechanical Systems (C-MEMS) has enabled the fabrication of three-dimensional glassy carbon nanostructures with geometries previously unattainable through conventional UV lithography. Pyrolysis of TPP structures conveys a characteristic reduction of feature size—one that should be properly estimated in order to produce carbon microdevices with accuracy. In this work, we studied the volumetric shrinkage of TPP-derived microwires upon pyrolysis at 900 °C. Through this process, photoresist microwires thermally decompose and shrink by as much as 75%, resulting in glassy carbon nanowires with linewidths between 300 and 550 nm. Even after the thermal decomposition induced by the pyrolysis step, the linewidth of the carbon nanowires was found to be dependent on the TPP exposure parameters. We have also found that the thermal stress induced during the pyrolysis step not only results in axial elongation of the nanowires, but also in buckling in the case of slender carbon nanowires (for aspect ratios greater than 30). Furthermore, we show that the calculated residual mass fraction that remains after pyrolysis depends on the characteristic dimensions of the photoresist microwires, a trend that is consistent with several works found in the literature. This phenomenon is explained through a semi-empirical model that estimates the feature size of the carbon structures, serving as a simple guideline for shrinkage evaluation in other designs.

## Carbon nanostructures: Determining post-pyrolysis physical properties

Linking carbon nanostructures’ pre- and post-shrinkage physical properties may help to design functional new materials. One nanomaterial fabrication technique involves the three-dimensional etching of a light-sensitive material, followed by heat degradation to shrink the structure to the nanoscale. Now, a global team led by Israel De Leon and Sergio Martinez-Chapa, from Mexico’s Tecnologico de Monterrey, have defined the relationship between a light-sensitive material’s original form and its post-heat-curing properties. Using the aforementioned technique, the team created a selection of carbon nanowires and found that their final physical properties depended on how the initial light-sensitive material was exposed to laser light, and its original surface area to volume ratio. Furthermore, thinner nanowires buckled under the high heat. From their results, the team created a model to estimate the metrics of carbon nanostructures and evaluate their shrinkage.

## Introduction

Carbon micro-electromechanical systems (C-MEMS) have emerged as a promising technology for the fabrication of miniaturized functional devices that used to be based almost exclusively on silicon^1^. C-MEMS are made up of carbon microelectrodes of a desired geometry, which are derived by patterning of a polymer precursor through photolithography, followed by thermal degradation using pyrolysis in an inert environment or vacuum. The thermal decomposition of ultraviolet (UV)-cured resins at high temperatures^2^ (~900 °C) is typically accompanied by a large volumetric shrinkage of up to 90%^3^, leading to smaller carbon structures with high aspect ratio. The pyrolytic process results in an sp^2^-rich form of carbon known as glassy carbon, which exhibits high isotropy, a wide electrochemical stability window, biocompatibility, superior chemical resistance, and under certain conditions, semiconductor-like electrical properties^2,4^. These characteristics are advantageous for the manufacturing of sophisticated microdevices, which span electrochemical nanosensors^5^ to mechanical metamaterials^4^.

In C-MEMS, the selection of the polymer manufacturing method plays a key role in the final geometry, dimensions and properties of the obtained carbon part^6^. Conventionally, UV lithography can be used to pattern a flat, two-dimensional layer of a photoresist template, which is subsequently converted into the desired carbon^7^. Its linewidth resolution is fundamentally limited by diffraction, and thus carbon electrodes are typically around 1 μm in width^8,9^, or slightly in the submicron range^10^. Ideally, the selected polymer manufacturing method should provide precise patterning, a tunable linewidth resolution to meet the demand for increasingly smaller devices, and a reasonable freedom of design to suit different applications.

Of the existing three-dimensional (3D) photolithographic techniques, TPP remains the only optical technology capable of producing complex, computer-designed, photoresist structures with fabrication resolutions beyond the limit of diffraction^11^. In TPP, the nonlinear optical absorption of a photosensitive polymer layer induces a highly localized polymerization reaction, resulting in a blob-like structure referred to as a *voxel*. The dimensions of a voxel are defined by the properties of three main components: the optical system, the photoresist, and the photoinitiator^11–13^. This versatile microfabrication method has been employed to produce a variety of geometries and structures, ranging from 3D photonic crystal structures^14^, to mechanical metamaterials^4,15^, and even conductive nanowires^16^.

In spite of the growing amount and increasing complexity of geometries obtained by TPP, the materials used in the technique have been mostly limited to cross-linkable polymers^16^. The challenge is to manufacture functional devices through TPP that are made from materials that possess useful electrical, magnetic, optical, and chemical properties^17^. In view of this, recent studies have explored the combination of TPP with a high temperature pyrolysis step to create versatile glassy carbon micro/nanostructures. These include glassy carbon “nanosculptures”^18^, woodpile structures^19–23^, buckyballs^24^, atomic force microscopy tips^25^, 3D carbon electrodes^26^, and even metamaterials through carbon nanolattices^4^. The combination of the fabrication flexibility from TPP and carbonization offers new opportunities in several industrial applications by providing glassy carbon structures with almost arbitrary shape that exhibit superior chemical resistance, better wear and oxidation resistance, thermal stability, higher elastic modulus, and biocompatibility^4,22,25^. These advantageous properties enable applications in metamaterials, nanoelectromechanical systems, photonic crystals, and damage-tolerant lightweight materials^22^. For instance, lightweight glassy carbon mechanical metamaterials have been reported to possess improved strength-to-density ratios (1.2 GPa at 0.6 g cm^−3^), in part due to the material-strengthening effects that take place through microlattice size reduction^4^. Moreover, the improved biocompatibility and mechanical strength of glassy carbon microstructures can be readily used for transdermal drug delivery, offering better stability and stiffness than most polymers^27^. In addition to these advantages, photoresist-derived glassy carbon has been used to fabricate conductive microstructures that have ohmic contacts^3,28,29^—a desirable feature in functional microdevices. Despite the importance in analyzing the inherent volumetric shrinkage experienced during pyrolysis of TPP structures, no studies to date have experimentally and theoretically assessed how the different geometrical building blocks, such as nanorods or micro/nanowires^30^, and bars^4,24^, transform upon carbonization. Thus, estimation of the voxel linewidth percent reduction would enable the rational design of the carbonized version of photoresist templates, without necessarily undergoing extensive optimization.

In this work, we study the pyrolysis-induced shrinkage of structures consisting of suspended microwires of photocured resin (SU-8 2050). By varying the TPP exposure dose on the photoresist, the linewidth of the photoresist microwires was controlled in the range of 1250–1800 nm and subsequently reduced to 300–550 nm carbon structures through pyrolysis. This variation was achieved by tuning the scanning velocity and power of the laser, to produce a change in the exposure dose. We examined the degree of shrinkage through direct measurement using scanning electron microscopy (SEM), and studied the quality of the obtained carbon material through energy dispersive X-ray spectroscopy (EDS) and Raman spectroscopy. In addition, we developed a semi-empirical model that estimates the near-isometrical transformation experienced by TPP structures after pyrolysis. The model is built from calculations of the carbonized residual mass fraction found in several reports from the literature^3,4,24,31,32^. Using this empirical model, we estimate the residual mass of the TPP structures, assuming that the volatilized material: (1) is solely lost to degassing; and (2) is dependent on the initially available surface area of the microstructures. The results from this empirical model reinforce the hypothesis that the overall mass loss process that occurs during pyrolysis is largely dependent on the surface-to-volume ratio of the initial photoresist microstructure, consistent with previous studies of C-MEMS^31^. The current approach fundamentally differs from other works presented in the literature in that we present: (1) the approximate functional form of the dependence between TPP exposure dose and feature linewidth after pyrolysis; (2) a model that relates the feature percentage reduction to the mechanical elongation and degassing of the structures; and (3) an empirical model that uses fitting parameters with a precise physical meaning, which allow its comprehensive application to other works and contexts. A good agreement was obtained between our model and the experimental linewidth measurements of the fabricated structures. Furthermore, we have found that nanowire structures with high aspect ratio (length over linewidth of 30 or more) can be susceptible to buckling, resulting in deflections in the range of 100–250 nm. By applying Euler’s buckling theory, we show that this behavior is likely caused by the thermal stress in the pyrolysis process. These results, together with the shrinkage predictions, serve as simple design rules that can help estimate carbon nanowire feature size and shape after the thermal deformation induced by high temperature pyrolysis.

## Results

### Fabrication of TPP structures

**Fig. 1:**
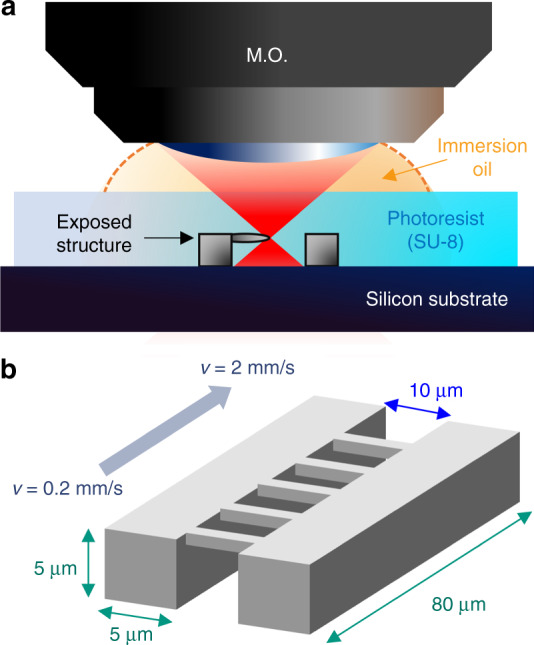
**a** Femtosecond laser exposure scheme used to fabricate suspended SU-8 wire structures, featuring the microscope objective (M.O.) coupled to the SU-8 2050 film using an immersion oil (N.A. = 1.4). **b** Schematic representation of the geometry of the exposed structure. The suspended wires are written at different speeds as indicated

We first undertook the fabrication of SU-8 microwires by varying scanning velocity (*v*) and laser average power (*P*_t_) to study the effect of dosage on voxel linewidth. Suspended SU-8 microwires were fabricated using a vertical exposure configuration, as depicted in Fig. 1a. The layered structure consisted of an unexposed photoresist layer (SU-8 2050) atop a pyrolysis-resistant silicon substrate. Microwires were then written by scanning the focal spot of the microscope objective into the photoresist volume, using an immersion oil droplet directly on top of the photoresist interface. In Fig. 1b, we illustrate a schematic representation of the exposure geometry, which consisted of parallel walls bridged by the suspended wires. Each microwire was written using a different configuration of *v* and *P*_t_, effectively making a dosage test field.

Following the fabrication of the suspended SU-8 microstructures, the samples were pyrolyzed in a furnace at 900 °C to obtain the carbon nanowire structures. The pyrolysis process consisted of a heat ramp of 5 °C/min up to 300 °C and then to 900 °C, with a dwell time of 1 h at each of those temperatures, followed by cooling to room temperature at approximately the same ramp. These values have been selected on the basis of previous works on the fabrication of C-MEMS from SU-8 precursors^7,31^. Briefly, the first dwell time temperature (300 °C) was selected because it corresponds to the critical point of thermal stability for the SU-8 2000 series (5%wt. loss starts slightly above 300 °C according to Microchem in the SU-8 2000 datasheet). In addition, leaving the sample for 1 h at this temperature allows the resin to slowly initiate the degassing process, while evacuating any residual oxygen from the chamber. In the case of the dwell time at 900 °C, we selected that configuration because previous analysis of Thermogravitometric Analysis (TGA) data shows that this is the point where the weight loss vs. temperature curve starts to decrease significantly in slope^31,33^. Furthermore, this final temperature has been associated with the effective carbonization degree of the sample^34^.

**Fig. 2:**
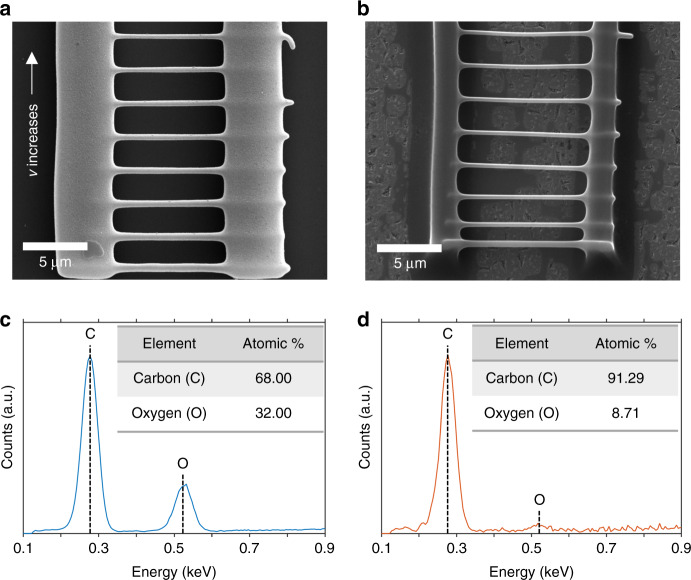
**a** Top view SEM image of SU-8 microbridge structures fabricated via TPP using different scanning velocities. **b** Top view SEM image of a pyrolyzed structure, showing the morphological change in linewidth and length of the wires. **c**, **d** EDS spectra with the corresponding carbon and oxygen atom peaks in structures (**a**, **b**), respectively

Photoresist microwires were visually inspected before and after their carbonization using SEM. In Fig. 2a, an SEM micrograph of a nonpyrolyzed TPP photoresist sample is presented. This microstructure represents a test field in which the scanning velocity was varied in the vertical direction, and the laser power was fixed. We observed that the TPP threshold for polymerization and successful patterning of the wires was 1.13 ± 0.12 mW, at a scanning velocity of 0.2 mm/s. Upon pyrolysis, photoresist microwires shrank and were converted into glassy carbon, both by losing heteroatoms to volatilization and by reconfiguration of the remaining carbon atoms in their structure.

### Characterization of carbonized TPP structures

To confirm the degree of carbonization of the samples, we analyzed their carbon-to-oxygen (C/O) weight percentage, before and after pyrolysis using EDS. The C/O content is commonly employed to characterize the composition of pyrolyzed samples^35^. Fig. 2c shows the high C/O content of the photoresist precursor, which is roughly in a ratio of 2:1. In Fig. 2c, d, the EDS spectra near the carbon peak (0.277 keV) is plotted for the photoresist and pyrolyzed material, respectively. EDS analysis revealed a 68.00% carbon content prior to pyrolysis, which transforms into a normalized carbon atomic percentage of 91.29%. Likewise, oxygen content, associated with the peak at 0.525 keV, changed from 32.00 to 8.71%, suggesting a high degree of heteroatom volatilization in the samples.

**Fig. 3:**
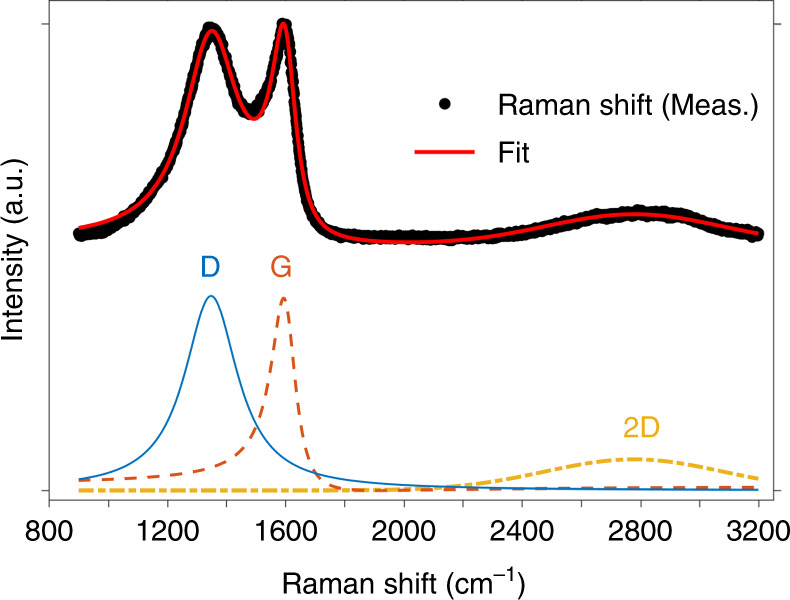
Measured Raman spectrum of the glassy carbon material derived from pyrolysis of TPP structures, along with its functional fit. The peaks have been deconvoluted to indicate the contributions from the D, G, and 2D bands, respectively

Pyrolysis of photoresists typically results in carbon materials that are glassy in nature. To confirm this, we examined the bulk structure of the samples by Raman spectroscopy. In Fig. 3, we show the spectrum of a supporting wall structure, rather than a nanowire, since the spot size of the visible excitation laser was not small enough to individually resolve the suspended carbon nanowires. This spectrum features three notable peaks, which have been deconvoluted for analysis: (1) the peak near 1594 cm^−1^, related to the graphitic G-peak (~1581 cm^−1^), that is normally attributed to bond stretching of all pairs of sp^2^ atoms^36^; (2) the peak at 1348 cm^−1^, indicative of the D-peak (~1360 cm^−1^), ascribed to the breathing mode of sp^2^ aromatic rings and structural defects^36,37^; and (3) a small modulated bump (~2400–3100 cm^−1^) near the 2D-peak (~2700 cm^−1^), which comes from an ill-defined second order of the D-peak^38^.

To deconvolute the spectrum in Fig. 3, we have fitted the data using the Breit–Wigner–Fano (BWF) line for the G-peak, which accurately describes the band asymmetry of amorphous carbons towards lower wave numbers^36^. The D and 2D peaks have been, respectively, fitted to a Lorentzian, which lies within the BWF family of curves, and a Gaussian, which accounts for the broadening of the second order Raman peak. In our samples, the G-peak is upshifted close to 1600 cm^−1^, possibly indicating the merging of G and D′ peaks due to high disorder^38^ or the presence of a small crystallite size^39^. Moreover, this low-range order is characteristic of a small in-plane correlation length (*L*_a_). For our samples, we find that *I*(*D*)/*I*(*G*) = 1.04, and calculate that *L*_a_ ~1.4 nm, according to the theory presented by Ferrari et al., which postulates the dependence $I\left( D \right)/I\left( G \right) \propto L_{\rm{a}}^2$^36^. The glassy nature of the sample is further confirmed by the complete absence of a doublet structure in the 2D peak, and along with a broad FWHM. The obtained FWHM for the 2D line is roughly 707 cm^−1^, representing a ~28-fold increase from that of single-layer graphene. Thus, 2D-peak broadening evinces of the absence of *c*-axis order in the samples^38^. Together with the estimation of *L*_a_, these observations provide a qualitative description of the microstructure present in the pyrolyzed sample.

**Fig. 4:**
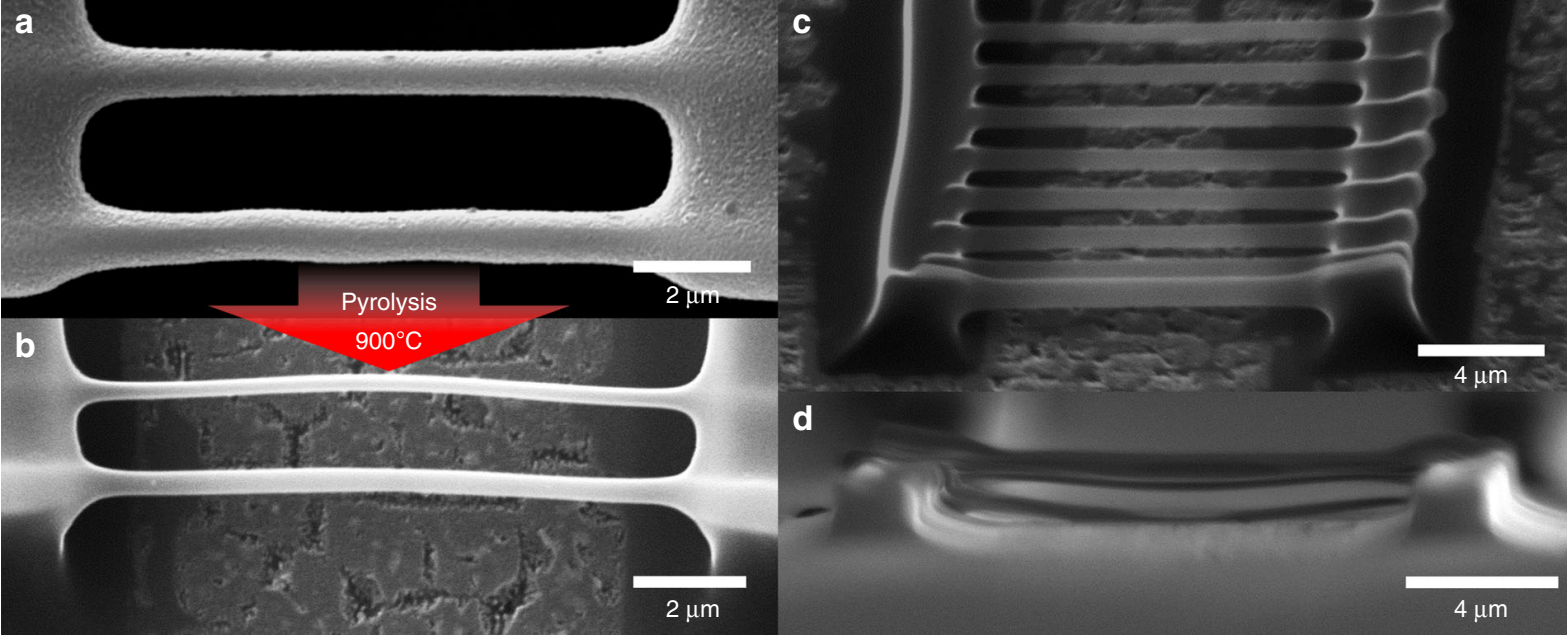
Close-up, top view of a pair of suspended SU-8 photoresist microbridges produced by TPP (**a**) before and (**b**) after pyrolysis at 900 °C. **c** Perspective view (65°) of a structure with carbon nanowires. **d** Side view of suspended carbon nanowires

The morphological evolution of the SU-8 wires is revealed in the change from Fig. 4a to their carbonized version in Fig. 4b. During pyrolysis, wires tend to thin down due to a degassing process, causing a volumetric shrinkage and therefore, linewidth reduction^3,40^. Depending on the configuration of the supporting structures before undergoing carbonization, the suspended photoresist can either stretch^40^ or contract^41^ in the axial direction. Thus, in order to estimate the elongation that the suspended photoresist wires would experience after pyrolysis, we first analyzed the shrinkage that the supporting anchoring points without the wires. In Fig. S2, we show an SEM micrograph of the side view of the supporting walls, which evinces how the separation between the anchoring points changes after pyrolysis, providing the pulling force that results on the axial deformation. The axial strain, given by *δ*[%] = (*L*_f_ − *L*_i_)/*L*_i_, was measured in our experiments as *δ*[%] = 14.1 ± 2.0, corresponding to wires stretching from an original length of *L*_i_ = 9.80 ± 0.40 μm to a final length *L*_f_ = 11.18 ± 0.22 μm. In the theoretical model, we will see that this stretching accounts for only a slight diameter shrinkage, but that is nonetheless required to get an accurate prediction of the linewidth reduction. Moreover, we have also obtained a perspective view (taken at 65°) of the pyrolyzed wires in Fig. 4c, as well as a side view SEM micrograph of the suspended carbon nanowires, in Fig. 4d. Using the side view images, we managed to image a few samples (outermost wires), capturing the characteristic voxel asymmetry. Upon measurement, we determined that the voxel aspect ratio (voxel axial length/linewidth) of the wires lies between 1.5 and 1.9, for the thinnest and thickest structures, respectively.

We observed that the thinner carbon nanowire samples were propense to buckling after the high temperature pyrolysis process, as seen in Fig. 4b. Hence, to provide a quantitative description of this deflection, we registered its magnitude using the obtained SEM micrographs (measured as the maximum deviation of the beam from the central axis). Afterwards, we compared the measured value against the aspect ratio of the nanowires; the comparison and significance of this curve is discussed in a later section of the paper.

### Theoretical model of voxel linewidth

An expression for the resolution of the TPP linewidth before pyrolysis can be obtained by taking into account the different parameters involved in the fabrication process, such as scanning velocity, average laser power, repetition rate, and pulse duration, τ_L_^42^:

$$d_{line} = \alpha \,w_0\left[ {\ln \left( {\frac{{\sigma _2N_0^2D}}{C}} \right)} \right]^{1/2}$$

1 $$C = \ln \left( {\frac{{\rho _0}}{{\rho _0 - \rho _{{\mathrm{th}}}}}} \right),\sigma _2 = \bar \sigma ^{\left( 2 \right)}\eta ,N_0 = \frac{2}{{\pi w_0^2\tau _Lf_{{\mathrm{rep}}}\hbar \omega _L}},$$

where *σ*_2_ is the effective two-photon cross section for the generation of radicals, defined by the product of the two-photon cross section, $\bar \sigma ^{\left( 2 \right)}$ and the efficiency of the initiation process^43^, *η* < 1. *N*_0_ is the maximum photon flux at the focal plane, described by a gaussian distribution^43^ with waist given by the lateral resolution of the focal spot, *w*_0_. The *f*_rep_ is the repetition rate of the laser, and *ω*_*L*_ = 2*πc*/*λ*_*L*_. The *C* is an integration constant given by the initial concentration of photoinitiator molecules *ρ*_0_ and the minimum concentration (threshold value) of radicals needed to polymerize the resin, *ρ*_th_; and *α* is a constant reflecting the characteristics of the exposure scheme, which describes the fact that diffraction limit is not the sole factor determining voxel feature size^44^. In writing Eq. (1), we have used the term *D*, which can be thought of as a TPP exposure dose in the scanning line configuration given by

2 $$D = f_{{\rm{rep}}}\tau _{L}\frac{{\sqrt \pi w_0}}{{2v}}P_{\rm{t}}^2$$

where *v* represents the velocity of the moving stage (scanning velocity), typically in µm/s to mm/s (see supplementary material for details). As seen from Eq. (2), the TPP exposure dose includes an inverse velocity dependence, which is proportional to the exposure time in analogy to the normal exposure scheme^42,43^. Note that the TPP exposure dose does not have energy density units as the conventional exposure dose (typically given in mJ/cm^2^), but rather is an experimentally determined parameter that condenses all the conditions used to perform the exposure experiments. Furthermore, *D* will have the same units (W^2^s) as in the case single voxel exposure^43^—where instead of using *v* and *P*_t_, one uses exposure time (typically in ms by controlling a shutter) and *P*_t_. Thus, given a set of experimental conditions, *D* can be defined and voxel linewidth will be fundamentally determined by this parameter as indicated by Eq. (1). However, for comparison purposes, *D* can be compared with the conventional single-photon exposure dose, as this last quantity is defined by exactly the same experimental variables (see supplementary material for details on this expression).

**Fig. 5:**
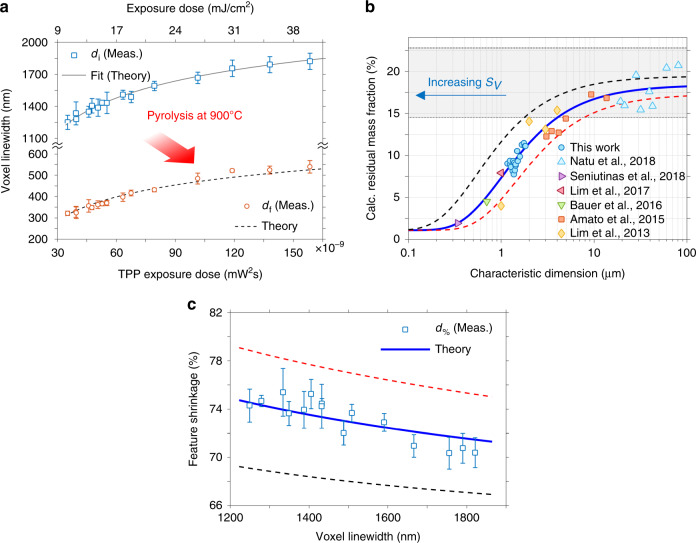
**a** Voxel linewidth variation with TPP exposure dose in the scanning line configuration. The continuous line represents the fitting of the voxel linewidth before pyrolysis (square markers); the dashed line shows the fitting of linewidth after pyrolysis (circle markers). **b** Calculated residual mass fraction after pyrolysis for several works reported in the literature and the current work, for cylindrical posts and suspended bar geometry [using ${\boldsymbol{w}}_{\boldsymbol{f}} = \left( {\frac{{{\boldsymbol{\rho }}_{\boldsymbol{f}}}}{{{\boldsymbol{\rho }}_{\boldsymbol{i}}}}} \right)\left( {1 \pm {\boldsymbol{d}}_{\boldsymbol{\% }}} \right)^2\left( {1 \pm {\boldsymbol{\delta }}_{\boldsymbol{\% }}} \right)$, see supplementary material]. The continuous line represents the fitting of the proposed mass fraction function, and the dashed lines corresponds to the 90% confidence intervals. **c** Feature linewidth shrinkage as a function of the initial voxel linewidth, along with the predicted value by the theory. The 90% confidence intervals have been added as dashed lines

We proceeded to plot Eq. (1) for the experimentally obtained voxel linewidths in Fig. 5a (square markers, *d*_i_) using *σ*_2_/*C* and *α* as least square fitting parameters. In this plot, we present the dependence of the linewidth of SU-8 wires with the TPP exposure dose as a continuous plot line, which is in agreement with previous reports on TPP of wires^30,45^. For comparison purposes, the secondary *x*-axis has been replaced with the exposure dose (mJ/cm^2^). However, notice that the spacing of this secondary axis is not linear, since the primary *x*-axis depends on ~$P_{\rm{t}}^2$, rather than on *P*_t_. By fitting the voxel linewidth data, we find that *σ*_2_/*C* = 2.92 × 10^−51^cm^4^ s and *α* = 3.16, which results in a coefficient of determination of *R*^2^ = 0.988. The obtained value for *σ*_2_/*C* closely resembles that reported in other works for SU-8, which place *σ*_2_/*C* ~3.00 × 10^−51^ cm^4^ s^42^. In our case, SU-8 2050 contains 3.4 wt.% of photoinitiator molecules (*ρ*_0_ = 3.4%) according to the vendor (Microchem) datasheet. Using typical values for the polymerization threshold (*ρ*_th_ = 0.25%), we calculate that *σ*_2_ = 2.23 × 10^−52^ cm^4^ s. This value represents a lower two-photon absorption sensitivity than those of specially designed initiators, such as *π*-conjugated molecules (~10^−50^–10^−47^ cm^4^ s)^43^, as expected. Last, other reports on the study of TPP structures place *α* around 2.88^37^, which is close to the obtained value in our fit.

### Theoretical model of voxel linewidth shrinkage

Next, we estimate the linewidth reduction after pyrolysis due to the stretching and degassing process. Note that a complete model that accurately predicts the morphological evolution of a photoresist wire due to pyrolysis would require at least the incorporation of interfacial and surface energies of each of the involved components (polymer, volatiles, and carbon), as these parameters significantly affect the shrinking process of the micro/nanostructures^46^. Nonetheless, if the residual carbon weight fraction [i.e. (*w*_f_ = *m*_f_/*m*_i_)] that remains after pyrolysis is known from previous experimentation, then one can estimate the linewidth reduction as follows. As a first approximation, we assume that the suspended photoresist bridges have a transversal circular cross-sectional area and total volume $V_{\rm{i}} = \pi d_{\rm{i}}^2L_{\rm{i}}/4$, where *d*_i_ and *L*_i_ are the initial diameter and length, respectively. Given that wires tend to stretch during pyrolysis in our experiments [see Fig. 4b], we assume that an axial deformation on a wire with Poisson’s ratio *v* induces a diameter reduction, according to the relation:

3 $$d^{\prime} = d_{\rm{i}}\left( {1 + \delta } \right)^{ - \nu },$$

where *d*′ is the diameter of the cylinder after the distortion (see the supplementary material for details)^47^. Although this reduction and that from the volatilization happen simultaneously during carbonization, we assume that the volumetric shrinkage can be modeled by first estimating the dimensions of the stretched cylinder [with diameter *d*′ and length *L*′ = (1 + *δ*)*L*_i_], and then factoring in the lost volume due to degassing. Thus, we note that the final volume will be given by a volumetric fraction of the volume prior to volatilization, i.e. *V*_f_ = *θV*′, where $\theta = \frac{{\rho _{\rm{i}}}}{{\rho _{\rm{f}}}}w_{\rm{f}}$ with *ρ*_i_ being the initial polymer density, *ρ*_f_ the resulting glassy carbon density, and *V*′ = *πd*′^2^*L*′/4. As an example, some references on the study of C-MEMS place *w*_f_ between ~0.15^33^ and ~0.30^31^, depending on the temperature of the pyrolysis treatment, heating rate, and polymer precursor. A derivation for the residual carbon mass fraction *w*_f_, and *θ* as a function of the initial geometry of the photoresist wires can be found on the supplementary material.

An important consideration in our simplified stretching model is that the *v* remains constant for the duration of the axial deformation. Strictly speaking, *v* will be a function of temperature and time, and possibly other variables. As the precursor material (SU-8) is progressively heated to temperatures beyond the glass transition temperature (*T*_g_), it enters a “rubbery” state prone to deformation^25^. However, studies on the dependence of the *v* of SU-8 with temperature indicate that it practically reaches a terminal value of ~0.5 near 200 °C^48^ (*T*_g_ = 215 °C according to the datasheet). Thus, since our experiments are carried out beyond this temperature, we have taken the value of *v* to be 0.5 as an approximation (which is the case of a perfectly incompressible material under elastic deformation).

In order to gain insight into the relationship between the residual mass fraction and the initial dimensions of the photoresist structures, we calculated *w*_f_ after pyrolysis for several cylindrical (or nearly cylindrical bars) photoresist structures and features reported in the literature. We proceeded to compare these points with our obtained values for *w*_f_ and plotted the results on Fig. 5b, where the *x*-axis represents the characteristic dimension (diameter) of the cylindrical structures. Although some of these works studied structures that are only geometrically approximate to our suspended bridges, they were chosen on the basis of their similarity to our samples in terms of: (1) the photolithography method used to derive them, i.e. UV-photolithography and TPP lithography; (2) the pyrolysis conditions used to fabricate them (~900 °C); (3) their aspect ratios (~10:1 or more); and (4) their surface area ratio (*SAR*) value^31^, where we have selected structures whose *SAR* *>* 1, meaning that the lateral surface of the wires was greater than their top surface (for cylindrical posts, common in C-MEMS) or their transversal area (for suspended bars). In the plot, we observe that the wealth of the data points to a reduction in *w*_f_ as the characteristic dimension reduces (or alternatively, as the surface-to-volume ratio *s*_*V*_ increases). This trend has been identified by a fit in the continuous line (see supplementary material for details on the equation); in addition, the functional fit has been calculated solely from the values found in the literature. Moreover, Fig. 5b shows how *w*_f_ increases with *d*_i_, reaching a terminal mass fraction value *w*_1_, enclosed by the boundaries of the grayed-out area of the plot. The upper and lower values of this range correspond to ~15%^33^ and ~23%^34^, which are the calculated remaining mass fractions after pyrolysis reported for spin-coated films of SU-8; this last number, however, can go as high as 27.2% according to other TGA measurements^49^. Clearly, our experimentally obtained values in the range of ~1–2 µm are in fair agreement with the overall trend of *w*_f_ vs. characteristic dimension. Therefore, the calculated values for *w*_f_ from the literature allowed us to establish a semiempirical relationship between initial geometry of the photoresist bridges and their residual mass after pyrolysis.

Once the residual carbon weight fraction and the remaining volumetric fraction were defined as a function of the characteristic dimension of the structures (*d*_i_), we combine the fact that *V*_f_ = *θV*′ with the contribution from Eq. (3), to show that the final diameter of the structure will shrink approximately according to the following relation:

4 $$d_{\rm{f}} = d_{\rm{i}}\sqrt \theta \left( {1 + \delta } \right)^{ - \nu },$$

where as before, $\theta = \frac{{\rho _{\rm{i}}}}{{\rho _{\rm{f}}}}w_{\rm{f}}$, and *w*_f_ = *f* (*d*_i_).

In Fig. 5a, the experimentally measured linewidths after pyrolysis are compared against the TPP exposure dose used to fabricate them. The dashed line corresponds to the theoretical value of *d*_f_ derived from Eq. (4) using *ρ*_i_ = 1.19 g/cm^3^^50^, *ρ*_f_ = 1.4 g/cm^3^^51^, *v* = 0.5^48,49^, and *δ* [%] = 14.1% (obtained from our measurements). For that plot, *d*_i_ was set to Eq. (1) using the previously found parameters. In addition, the function *w*_f_ = *f*(*d*_i_) was defined using solely the fitted parameters from the literature, which correspond to an effective mass flux of *j*_m*,* eff_ ~62 fg/(μm^2^ h), and residual fractions of *w*_0_ = 0.01, *w*_1_ = 0.18, where we have used an effective pyrolysis time of *τ*_p, eff_ ~4.9 h as in our experiments (see supplementary material for details). Therefore, the plot from Fig. 5a essentially represents a prediction of the pyrolyzed carbon nanowire diameter *d*_f_, for a given value of exposure dose. By comparing each experimental value of *d*_f_ to its predicted value, we obtained an *R*^2^ = 0.994, indicating that the experiments are in close agreement with the presented model.

Last, the results from Eq. (4) can also be summarized by indicating the feature shrinkage percentage for each photoresist wire of a given initial diameter, i.e., $d_\% \left( {d_{\rm{i}}} \right) = 1 - \frac{{d_{\rm{f}}}}{{d_{\rm{i}}}}$, or

5 $$d_\% \left( {d_{\rm{i}}} \right) = 1 - \sqrt \theta \left( {1 + \delta } \right)^{ - \nu } \cdot$$

Figure 5(c) shows the shrinkage percentage for each measured pair (*d*_f_, *d*_i_), illustrating the dependence of the reduction on initial geometry. In this graph, the continuous line represents the expected reduction predicted by the theory. Moreover, since the plot for *d*_%_ depends on *θ*, we have used the confidence intervals (90%) from Fig. 5b to estimate upper and lower boundaries for Fig. 5c. Although Eq. (5) provides a fair estimate of the feature shrinkage percentage, it is important to note that the obtained coefficient of determination indicated a slight deviation, with *R*^2^ = 0.71, which is clear from Fig. 5c. This deviation comes from the fact that *d*_%_ is a sensitive variable of *d*_i_, and thus slight differences in *d*_%_ against its theoretical value are expected to result in reduced *R*^2^ values, even though the maximum difference from the theory is only of 1.6% (for *d*_i_ = 1405 nm). In addition, the trend in the predicted graph would seem to indicate that the deviation from the theory increases as the wire linewidth increases. We believe the shortcomings in our model primarily stem from the assumption that voxel lines are axisymmetric. The thicker samples (1400–1800 nm) have been fabricated using a higher energy density dose, and thus are likely to present higher voxel asymmetry (the maximum voxel asymmetry of ~1.9 for *d*_i_ = 1821 nm). Hence, we hypothesize that longer voxel structures could result in more lateral area available for degassing, meaning more reduction, which could explain why our prediction falls short. Nonetheless, more experiments specifically studying the effect of voxel asymmetry are necessary to support this claim. Another plausible source of error could come from the predicted trend in Fig. 5b, which broadly assumes that the observed behavior in the literature is applicable to our devices. Different fitting values on that graph inevitably introduce a systematic error on Fig. 5c by shifting the predicted shrinkage curve up or down, as seen from the confidence interval dashed lines.

### Buckling of the carbon nanowires

**Fig. 6:**
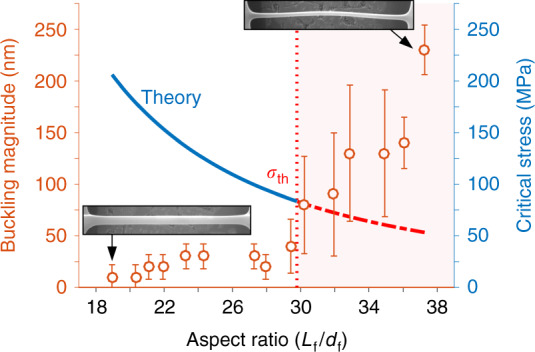
Buckling magnitude plotted against the carbon nanowire aspect ratio *L*_f_*/d*_f_. The secondary *y*-axis shows the theoretical critical stress obtained from the Euler buckling model. Stress values for which *σ*_c*r*_ < *σ*_th_ are indicated by the red shaded area

We now turn our attention to the buckling present in the thinnest samples of the carbon nanowires. In Fig. 6, we present a plot of the buckling magnitude (i.e., the distance from the central axis of the wire to the point of maximum deflection) vs. the aspect ratio of the pyrolyzed suspended structures (*L*_f_*/d*_f_. From the graph, it is clear that wires in the aspect ratio range 19 ≤ *L*_f_/*d*_f_ < 30 corresponding to 400 nm <*d*_f_ ≤550 nm experience negligible buckling, while those in the range *L*_f_*/d*_f_ > 30 (300 nm ≤ *d*_f_ <400 nm) tend to deflect significantly, with deflections of 230 ± 24 nm. A plausible explanation for the buckling can be proposed by first estimating the critical stress that a nanowire of a given aspect ratio (or slenderness ratio) can sustain before it bends, and then by comparing that value with the thermal stress induced by the pyrolysis process. For a wire of a given *d*_f_, *L*_f_, and Young’s modulus *E*, pined on both sides, the critical stress will be^52^:

6 $$\sigma _{cr} = \frac{{\pi ^2E}}{{\left( {\frac{{KL_{\rm{f}}}}{{r_{\rm{g}}}}} \right)^2}},$$

where *K* is the effective length factor (*K* = 0.5 for a pined-pined case), and *r*_g_ is the radius of gyration (*r*_g_ = *d*_f_/4, assuming a circular cross section). In the secondary axis of Fig. 6, we have plotted Eq. (6) as a continuous line using *E* = 30 GPa^53^. Last, we have plotted a vertical dashed line that represents the aspect ratio value at which *σ*_cr_ = *σ*_th_, which we have defined as *σ*_th_ = *Eα*_L_(*T*_0_−*T*_f_), where *α*_L_ is the coefficient of thermal expansion in [1/K], and *T*_0_,*T*_f_ represent the initial and final temperatures in the heating/cooling process. From Fig. 6, we observe that wires with lower critical stress than *σ*_th_ ~84 MPa exhibit the greatest buckling. This is a plausible value for *σ*_th_ if we assume Δ*T* ~875 °C (difference with room temperature of 25 °C), resulting in *α*_L_ ~3.2 × 10^−6^ °C^−1^, which is within the reported figures for glassy carbon^6,54^. In Fig. 6, we see that a stress of *σ*_th_ can be withstood by a wire with aspect ratio of ~30 according to the theory—a reasonable threshold, since wires with greater aspect ratio present significant buckling.

## Discussion

Shrinking values herein reported range between 70 and 75%, in agreement with previous reports on the pyrolysis of suspended SU-8 structures^40^. This reduction is likely caused predominantly by volatilization losses, rather than strain induced effects. For instance, the assumption of no degassing in Eq. (5), (*w*_f_ = 1), would result in *d*_%_ ~16%, which does not match our experimental observations. This argument is further substantiated by the fact that a significant strain would likely result in a noticeable necking deformation in the central part of the nanowires. However, we found that linewidths did not exhibit a significant variability in the axial direction [see Fig. 4b]. From the plot on Fig. 5c, it can be seen that pyrolysis of the samples resulted in a volumetric shrinkage dependent on the initial linewidth of the suspended microwires, with thinner samples leading to the greatest shrinking values. This observation suggests that *w*_f_ is linked to the size of TPP structures due to the dependence of volatilization with the available surface interface^31^, which is greater in smaller samples because of their increased surface-to-volume ratio. Therefore, the overall hypothesis that shrinkage is dependent on surface-to-volume ratio is supported by our results and the pattern found for previous studies, as demonstrated in Fig. 5b.

Note that while *SAR* values have been used in the past to assess its effects on volume shrinkage, to our knowledge, this has only led to empirical estimations that entail no direct physical interpretation^31^. In contrast, the expression that we give for the residual mass fraction after pyrolysis specifically gives insight into to the important parameters that affect it *(i.e., j*_m, eff_, *τ*_p, eff_, *ρ*_i_*)*. Thus, the model takes as input the information from the polymer wire geometry, physical properties (*v*, material densities *ρ*_i_, *ρ*_f_), and the surface-to-volume vs. calculated residual mass fraction behavior (obtained from the literature), in order to produce the theoretical estimation. Once obtained, this estimation can be compared with the experimentally obtained postpyrolysis linewidths, rather than performing a function fitting the data, thereby facilitating the optimization and rational design of the desired carbon nanowire structures.

The presented fabrication scheme has several advantages for the fabrication of suspended carbon nanowires. By using a carbon rich precursor with sufficient mechanical robustness like SU-8 2050, we were able to create freely suspended carbon structures that did not collapse upon carbonization. Such problem has been reported particularly with different photoresists, as is the case of IP-Dip used in commercial TPP setups^25^. Choosing pure SU-8, however, comes with the disadvantage of lower *σ*_2_ sensitivity, and therefore bigger linewidth features, because it contains only conventional UV-absorbing cationic photoinitiator molecules (triarylsulfonium hexafluorantimonium) with a single photon absorption peak at 365 nm^45^. Another remarkable result is that the dosage dependence of Eq. (1) is retained even after the deformation induced by the high temperature pyrolysis, as shown in Fig. 5a (circle markers, *d*_f_). This allows for acceptable control over the final dimensions of the nanostructures in the range 300–550 nm. Photoresist nanostructures subjected to carbonization are known to suffer semi-isotropic shrinkage, characterized by “gum-like” distortions in one or several directions, depending on the topography of the substrate they are fixed to^4^. Nonetheless, we believe that the SU-8 line structures here presented suffered a marginal deformation of this kind, as the height of the structures prior to pyrolysis was relatively short (5 µm), and the effect is known to become significant for tall structures only (20–80 µm, or more)^40^.

Although TPP allowed us to create suspended 3D carbon nanowires, the objective of this study was not to exploit the versatility of the technique to create a complex geometry. Rather, the main objective was to systematically analyze how the suspended structures written by TPP transform upon carbonization. Nonetheless, a notable advantage of these structures is that they are seamlessly embedded within two posts in a single piece (support wall-wire-support wall). This “monolith” configuration is expected to result in a highly stable ohmic contact^3^, which can be straightforwardly used as a platform to produce nanogap-sized electrodes^28^. In this prospect, the combination of TPP with pyrolysis is expected to be particularly useful, since it can routinely provide a fine control over nanowire length, and hence, over nanogap size^28^.

Other possible C-MEMS applications that benefit from the manufacturing of carbon nanowires with predictable feature sizes include label-free impedance-based nanobiosensors^55^, nanoparticle-based gas sensors^56^, and electrochemical immunosensors^5^. In these perspective applications, the multiple pyrolysis parameters (i.e., flow rate, heating/cooling ramp rate, intermediate/final temperatures, and dwell times) are expected to play a decisive role over the electrical, physical, and electrochemical properties of the derived glassy carbon^57^. However, in this introductory study, we decided to maintain the pyrolysis conditions fixed in order to focus on the impact of the prepyrolysis geometry on feature shrinkage. This reasoning is substantiated by the fact that feature shrinkage primarily depends on initial geometry, when compared with other pyrolysis parameters^31^. Thus, future studies could include not only the effect that flow rate, ramp rate, or other pyrolysis parameters have on shrinkage, but also the influence they have on the required physical and chemical properties of the manufactured C-MEMS device.

Contrary to our methodology, other available technologies that integrate carbon nanowires into C-MEMS structures conventionally require a two-step process consisting of (1) the fabrication of the micron-ranged supporting structures (usually through UV-photolithography) and (2) the fabrication of the carbon nanowire precursor, for example through two-step photolithography^58^, far field electrospinning^29,55^, or electromechanical spinning^40^. Leveraging the fabrication advantages of TPP, however, comes with added fabrication complexity, in terms of required equipment and operating conditions (i.e., a stable femtosecond pulsed laser). Moreover, the voxel asymmetry (not studied here), will likely result in carbon nanowires with noncircular cross sections. Despite these shortcomings, the scanning voxel configuration provides a fine, two-parameter control (*v* and *P*_t_) over feature linewidth, which remains to be rivaled by more commonly used electrospinning-based methods.

## Conclusions

The present study provides a methodology to evaluate the morphological and geometrical dependence of carbon nanowire/C-MEMS hybrid structures with TPP process variables (i.e., scanning velocity, laser power, repetition rate, and pulse duration). Furthermore, the presented analysis showed how the volumetric reduction in the carbon nanowires can be directly linked to the increase of surface-to-volume ratio in the structures. Although this connection had been previously pointed out, our semi-empirical model and experimental results elucidate the relationship in more quantitative terms. We believe these guidelines could provide an effective method for the optimization of more complex C-MEMS micro/nanostructures produced by TPP.

The presented model for the TPP linewidth resolution, however, contains assumptions that could be improved in future works, such as the voxel diameter versus voxel length symmetry^43^ and the simplification of the pyrolysis volatilization mechanics. More topologies of the wire-supporting structures could be explored to induce different deformations upon pyrolysis. Depending on the intended application, a careful microstructure analysis will be required, for instance, conductivity measurements in the case of carbon microwire impedance-based sensors. In performing those studies, the design versatility provided by TPP will facilitate the integration of the template photoresist structure within intricate geometries, which would allow for insightful microstructure characterization.

## Materials and methods

### Photoresist film preparation

The photoresist structure preparation began with polymer coating of the silicon substrate. Silicon squared substrates (Si, 2 cm × 2 cm, thickness 525 µm) were cleaned using a 5-min acetone ultrasonic bath, followed by 5-min rinsing with isopropyl alcohol under the same conditions. Substrates were then taken to an oxygen plasma treatment for 2 min for further cleaning. Afterwards, SU-8 2050 (Micro Resist Technology GmbH, DE) was spin coated for 10 s at 5000 rpm (accel: 100 rpm/s) followed by 30 s at 3000 rpm (accel: 300 rpm/s) to evenly spread a photoresist thin film. Using these specifications, a 43-μm-thick film was obtained, which was measured using a Bruker Dektak XT profilometer. Next, softbake of the film was carried out at 95 °C for 8 min. Samples were then taken to the TPP setup for exposure. Following this step, postexposure bake of the samples was performed at 95 °C for an additional 8 min to complete the cross-linking process. Finally, samples were taken to developing using SU-8 developer (mr-Dev 600, Micro Resist Technology GmbH, DE) for 8 min in sonication.

### TPP writing procedure

The TPP experiments were carried out using a previously reported custom-made setup^59^. TPP was obtained using a femtosecond pulsed laser (Coherent Mira 900D titanium:saphire laser) with central wavelength of *λ* = 800 nm and a pulse duration of *τ*_L_ = 150 fs, at a repetition rate of *f*_rep_ = 76 MHz, pumped by a frequency-doubled Nd:YVO_4_ laser (Coherent Verdi V10). The resonator was set for practical purposes its most efficient wavelength (800 nm). This value closely corresponds to those which have been used in previous TPP experiments with SU-8 resins^30,45,60,61^. In addition, it has been previously shown that off-the-shelf SU-8 has a good light intensity threshold for two-photon absorption close to that wavelength (796 nm)^45^. The laser beam was coupled to a conventional upright optical microscope (Zeiss Axioplan) featuring a Zeiss Apochromat 100× oil immersion objective lens (NA = 1.4). Before entering the optical microscope, the laser beam was expanded to fill the back aperture of the objective lens in order to achieve a lateral resolution of *w*_0_ ≈0.61*λ*/NA = 350 nm.

A three-axis nanometer positioning stage (PI P-563.3CD) was used to scan a focal spot into the photoresist sample. In addition, an automatic shutter (Thorlabs SC10), a motorized gradient neutral density filter, and a photodiode (Thorlabs DET 110) were used to control exposure time and transmitted power of the laser.

In order for samples to be fixed to the substrate [Fig. 1a], the vertical region where SU-8 lies must be manually located by monitoring the intensity of fluorescence with a CCD camera, while varying the focal distance to the sample. This fluorescence must be induced with a low laser power (0.8 mW in our case) to prevent polymerization prior to fabrication start up. When the focal spot was within the immersion oil, no characteristic fluorescence response was recorded. However, once the focal spot reached the SU-8 interface, the fluorescence readout was evident, followed by a fully measurable fluorescence peak when penetrating 1–2 µm into the sample. After further movement of the focal spot the fluorescence signal eventually disappeared, indicating that the silicon/photoresist interface was found. This procedure was repeated two more times at relative coordinates to define a three-point plane (of 300 µm^2^) which described the orientation of the sample.

A total of 15 pairs of walls were produced per silicon substrate, varying *P*_t_ from 0.2 to 3 mW in steps of 0.2 mW, corresponding to peak powers from 17.5 to 263 W. The walls were 5 µm tall, 5 µm wide, and 80 µm long, and the separation between them was fixed to 10 µm [Fig. 1b]. The number of wires for each pair of walls was set to 19, varying the scanning velocity from 0.2 to 2 mm/s in steps of 0.2 mm/s.

### Pyrolysis of TPP structures

Samples were pyrolyzed in a furnace (PEO 601, ATV Technologie GmbH, DE) at 900 °C. In order to create an inert environment to perform pyrolysis, the furnace was flooded by a continuous flow of ultra-high purity nitrogen (N2, 99.999%) at 5.5 L/min for 100 min. Afterwards, samples were heated at a rate of 5 °C/min up to 300 °C and then to 900 °C with a dwell time of 1 h at each of those temperatures. Finally, samples were cooled down to room temperature. A plot of the described protocol can be found in Fig. S3.

### Characterization

Visual inspection of the photoresist microstructures was carried through SEM, using an EVO MA25, Carl Zeiss AG microscope (Oberkochen, DE). Five linewidth samples were taken per wire to assess the dependence of voxels with TPP exposure dose. After carbonization, the linewidth of samples was evaluated again to measure the degree of shrinkage induced by pyrolysis. We analyzed the C/O ratio of these samples through EDS (XFlash 6, Bruker Corporation, Billerica, MA, USA). Due to the limited area that micro/nanowires offer for analysis, the EDS spectra was obtained and averaged from a supporting wall structure.

Pyrolyzed photoresist samples were characterized by Raman Spectroscopy using a Reinshaw InVia Raman Microscope (excitation laser: 532 nm), to evaluate the microstructure and graphitization within pyrolyzed samples. The spot of the laser was focused on the supporting structures (5 µm × 5 µm × 80 µm), which had an adequate size to resolve the Raman spectra. The entrance slit was set 65 µm, and the grating to 2400 I/mm grating, using a 12-pixel area.

## Supplementary information

Supplementary Material
