# Supplementary material for: Pyrolysis-induced shrinking of three-dimensional structures fabricated by two-photon polymerization: experiment and theoretical model
Source: Microsyst Nanoeng. 2019 Aug 26;5:38. doi: 10.1038/s41378-019-0079-9 (PMC6799819; doi:10.1038/s41378-019-0079-9)
Supplement: Supplementary file 1 — Supplementary Material [file 41378_2019_79_MOESM1_ESM.pdf]

Supplementary material

Pyrolysis-induced shrinking of three-dimensional structures fabricated by two-photon polymerization: Experiment and theoretical model

Braulio Cardenas-Benitez,[1] Carsten Eschenbaum,[2,3] Dario Mager,[3] Jan Korvink,[3] Marc J. Madou,[1,4] Uli Lemmer,[2] Israel De Leon,[1,*] and Sergio O. Martinez-Chapa.[1,*]

[1]*School of Engineering and Sciences, Tecnologico de Monterrey, Av. Eugenio Garza Sada 2501 Sur, Monterrey, NL, 64849, Mexico*

[2]*Light Technology Institute, Karlsruhe Institute of Technology (KIT), Engesserstraße 13, 76049 Karlsruhe, Germany*

[3]*Institute of Microstructure Technology, Karlsruhe Institute of Technology (KIT), Hermann-von-Helmholtz-Platz 1, Eggenstein-Leopoldshafen, 76344, Germany*

[4]*Department of Mechanical and Aerospace Engineering, University of California, Irvine, 4200 Engineering Gateway, Irvine, CA, 92697, USA*

Contents

1. Notes on the two-photon polymerization exposure scheme 2
- *a. Linewidth resolution in scanning writing configuration* 2
- *b. Single Photon Exposure in scanning writing configuration* 4

2. Wire elongation 5
- *a. SEM images of supporting walls* 5
- *b. Diameter change as a function of axial elongation* 5

3. Volumetric reduction after pyrolysis 6
- *a. Calculation of w_f from experimental data* 8

4. Pyrolysis protocol 9

5. References 9

1. Notes on the two-photon polymerization exposure scheme

a. Linewidth resolution in scanning writing configuration

We proceed to calculate the linewidth of a photoresist nanowire that can be created by scanning a Gaussian profile along a straight-line path.[1] We begin by writing the photon flux density profile $[\mathrm{photons}/(\mathrm{m}^2\mathrm{s})]$ of a traveling gaussian beam with waist w_0, which can be represented as

$$\bar{I}(x, y, z = 0, t) = \bar{I}_0 \exp(-2((x_c - x)^2 + (y_c - y)^2)/w_0^2) \qquad \text{(S1)}$$

where a constant photon flux $\bar{I}_0$ has been assumed, and (x_c, y_c) corresponds to the moving center of the profile. We assume a scanning movement only in one direction (y), so that y_c can be written as $y_c = v_y t'$ (see Fig. S1). The rate of change of the density of radicals as a function of position, $\rho(r, z, t)$, that is produced by the femtosecond pulses is proportional to the two - photon cross-sectional area [2]

$$\frac{\partial \rho}{\partial t} = (\rho_0 - \rho)\sigma_2 \bar{I}_0{}^2. \qquad \text{(S2)}$$

In Eq.(S2), ρ_0 is the initial density of radicals and $\sigma_2 = \bar{\sigma}^{(2)}\eta$ is the effective two-photon cross section for the generation of radicals, which is defined by the product of the two-photon cross section and the efficiency of the initiation process, $\eta < 1$.[2] Polymerization is defined to occur at any point within the resin volume where a minimum concentration threshold of radicals is achieved, i.e., $\rho(r, z) \geq \rho_{\mathrm{th}}$. An integration of Eq.(S2) yields:

$$C = \int_{\rho_0}^{\rho_{\mathrm{th}}} \frac{d\rho}{\rho_0 - \rho} = \sigma_2 \int_{-\infty}^{\infty} \bar{I}_0{}^2 \exp\left(-4\left(x^2 + \left(v_y t'\right)^2\right)/w_0^2\right) dt' \qquad \text{(S3)}$$

Notice that the limits on the right-hand side of Eq.(S3) are taken from $(-\infty, \infty)$ because, as seen from Fig. S1, the exposure at a single observation point depends on the integrated photon flux before and after the gaussian profile crosses that point. In writing Eq.(S3), we have assumed that the laser is continuously exposing the photoresist sample. However, because polymerization is achieved by the accumulation of radicals from the multiple femtosecond

pulses,[3] we approximate the right-hand side of Eq.(S3) to

$$C = f_{\mathrm{rep}} \tau_L \sigma_2 \int_{-\infty}^{\infty} \bar{I}_0^{\,2} \exp\left(-4\left(x^2 + \left(v_y t'\right)^2\right)/w_0^2\right) dt' \tag{S4}$$

where we have multiplied the integral term by the effective fraction of the time that the sample is being illuminated, which is given by the duty cycle ($f_{\mathrm{rep}} \tau_L$).

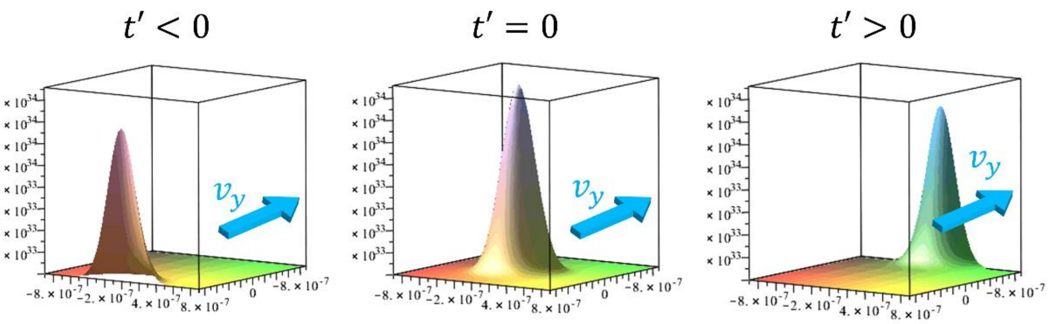

Fig. S1. Traveling Gaussian profile representing the magnitude of the photon flux, with movement in the y-axis for three different times of exposure. Adapted from thesis dissertation in ref. [1].

Neglecting the drop of radicals between laser pulses,[3] the solution to Eq.(S4) can be written as

$$C = \sigma_2 \bar{I}_0^{\,2} \exp\left(-\frac{d_{line}^2}{w_0^2}\right)\left(\frac{\sqrt{\pi} w_0}{2 v_y}\right)\left(f_{\mathrm{rep}} \tau_L\right) \tag{S5}$$

where have used the fact that $d_{line}^2 = 4x^2$, because we have set the threshold to be $\rho = \rho_{\mathrm{th}}$ at $2x = d_{line}$. Solving for d_{line} we obtain the following expression [4]:

$$d_{line} = w_0 \left[\ln\left(\frac{f_{\mathrm{rep}} \tau_L \sqrt{\pi} w_0 \sigma_2 \bar{I}_0^{2}}{2 v_y C}\right)\right]^{1/2}. \tag{S6}$$

However, Eq.(S6) can be simplified by introducing the two-photon exposure dose, given by

$$D = f_{\text{rep}}\tau_L \frac{\sqrt{\pi}w_0}{2v} P_t^2 \tag{S7}$$

where P_t is the average laser power, and $v_y = v$. In writing Eq.(S7), we have used the following notation:

$$\overline{I_0} = N_0 P_t \tag{S8}$$

where $N_0 = 2/(\pi w_0^2 \tau_L f_{\text{rep}} \hbar\omega_L)$.[3] Thus, combining Eq. (S6) and Eq.(S7), it can be seen that

$$d_{line} = w_0 \left[\ln\left(\frac{\sigma_2 N_0^2 D}{C}\right)\right]^{1/2}. \tag{S9}$$

Ultimately, Eq. (S9) can be complemented with a correction factor α, which is a constant reflecting the characteristics of the exposure scheme; this constant which describes the fact that diffraction limit is not the sole factor determining voxel feature size.[5] Therefore, we conclude that the voxel linewidth in the scanning line configuration can be written as

$$d_{line} = \alpha w_0 \left[\ln\left(\frac{\sigma_2 N_0^2 D}{C}\right)\right]^{1/2}, \tag{S10}$$

which corresponds to Eq. (1) in the main manuscript.

b. Single Photon Exposure in scanning writing configuration

In order to compare the values of two-photon exposure dose with more commonly used units of exposure (such as mJ/cm^2), we note that the single photon exposure dose (or exposure energy density) can be obtained in a similar fashion to the previous derivation.[6] Similarly to the two-photon exposure dose, the conventional exposure dose is dependent on the scanning velocity v, beam waist w_0, laser average power P_t, repetition rate f_{rep} and pulse duration τ_L. For a continuous laser exposure, the dose is given by:[6]

$$E_0 = \sqrt{\frac{2}{\pi}} \frac{P_t}{w_0 v}, \tag{S11}$$

Similarly, if we account for the effective time that the laser irradiates the sample due to its pulsation, the above expression becomes:

$$E_{0,\mathrm{eff}} = f_{\mathrm{rep}} \tau_L \sqrt{\frac{2}{\pi}} \frac{P_t}{w_0 v} \,, \tag{S12}$$

as a first approximation. We have used Eq. (S12) to compute the exposure energy dose in Fig. 5(a) in the main manuscript.

2. Wire elongation

a. SEM images of supporting walls

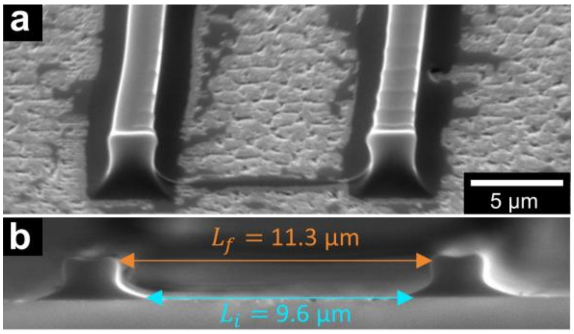

Fig. S2. (a) Angled-view of the supporting wall structures, after pyrolysis. (b) Side-view of the supporting wall structures, portraying the change in separation at the top-most part, where wires would be deposited.

b. Diameter change as a function of axial elongation

The definition of Poisson's ratio indicates that:

$$\nu = -\frac{d\varepsilon_{trans}}{d\varepsilon_{axial}} \,, \tag{S13}$$

where ε_{trans} is the transverse strain and ε_{axial} is the axial strain of a deformed material. For a cylinder stretched in the axial direction, an increase of ΔL will produce a reduction in

diameter in the transverse direction. From the Eq. (S13) , $d\varepsilon_{trans} = \frac{dx}{d_i}$ and $d\varepsilon_{axial} = \frac{dL}{L_i}$, where x is a dummy variable for diameter and L_i is the wire original length. Substituting these expressions into Eq. (S13) and integrating from $x = d_i$ to $x = d'$, and from $L = L_i$ to $L = (1 + \delta)L_i$, we that find:

$$1 + \frac{d' - d_i}{d_i} = (1 + \delta)^{-\nu} , \tag{S14}$$

where $\delta = (L_f - L_i)/L_i$. Or equivalently:

$$d' = d_i(1 + \delta)^{-\nu} , \tag{S15}$$

which is Eq. (3) on the main manuscript.

3. Volumetric reduction after pyrolysis

A photoresist microwire with a cylindrical shape will have two characteristic dimensions: diameter d_i and length L_i, with corresponding surface-to-volume ratio $s_V = \frac{4\pi d_i L_i}{\pi d_i^2 L_i} = 4/d_i$. Upon pyrolysis, this microstructure will begin to decompose by losing heteroatoms to gas evolution, resulting in a mass loss and a volumetric reduction. Mass loss can be described in terms of the remaining weight fraction after the process, namely $w_f = m_f/m_i$, and the remaining volumetric fraction is defined as $\theta = \frac{\rho_i}{\rho_f} w_f$. Therefore, to obtain θ, we require an expression for w_f. Two plausible hypotheses can be established for this quantity: 1) w_f (or θ) should be at least dependent on initial geometry, as demonstrated in the literature (see for instance ref. [7] and [8]); 2) w_f cannot be lower than the fraction of carbon mass that does not volatilize even after thermal decomposition. From the definition of w_f, it follows that

$$w_f = \frac{m_i - \Delta m}{m_i} = 1 - \frac{\Delta m}{m_i} \tag{S16}$$

where $\Delta m = m_i - m_f$, is the lost mass after pyrolysis. In processes involving volatilization of species, Δm will typically depend on the available surface for degassing (S) and mass flux (j_m). Although S will vary in time during the complete pyrolysis process, we assume that this

change will be slow (in the order of hours, in our pyrolysis protocol, see Fig.S3). An integration of the mass loss yields:

$$w_f = 1 - \frac{\int_0^{\tau_p} j_m(t)\, S(t)dt}{m_i} = 1 - \frac{\int_0^{\tau_p} j_m(t)\, S(t)dt}{\rho_i V_i} \quad \text{(S17)}$$

where the τ_p represents a time constant in the order of magnitude of the complete pyrolysis process. Eq. (S17) gives some insight into the geometrical dependence of w_f, since it decreases as the surface-dependent integral approaches $\rho_i V_i$. We therefore establish that this quotient is a function of s_V. This statement is plausible, as recent studies on the pyrolysis of cross-linked photoresists have found the direct and statistically significant relationship between shrinkage and surface area ratio (*SAR*),[7] which for cylindrical posts of height H and radius r is defined as

$$SAR = \frac{\text{Lateral surface area}}{\text{Top surface area}} = \frac{2\pi r H}{\pi r^2} = \frac{2H}{r} = H s_V \quad \text{(S18)}$$

where H is analogous to L_i in our analysis. Vertical cylindrical posts possess lateral and top surfaces available for degassing, and are therefore not equivalent to suspended photoresist wires,[8,9] which only have one lateral surface. However, they represent the one of the most studied cylindrical geometries in terms of shrinkage after pyrolysis.[7,10] Thus, we have used glassy carbon micro-posts arrays as our model to test our proposed shrinkage equation.[7]

Next, we introduce our main assumption, which consists in claiming that w_f cannot reduce indefinitely with arbitrarily increasing s_V. This hypothesis follows from our assumption 2), since there is an intrinsic amount of formed glassy carbon that cannot be lost to gas evolution for the considered pyrolysis conditions. If the rate of change of w_f decreases linearly as w_f approaches this intrinsic limit ($w_f = w_0$), we can propose the following differential equation for w_f:

$$\frac{\partial w_f}{\partial s_V} = -\beta\left(w_f - w_0\right), \quad \text{(S19)}$$

where β is a constant. The straightforward solution of Eq. (S19) yields

$$w_f = w_0 + (w_1 - w_0)\exp(-\beta s_V), \quad \text{(S20)}$$

which has three parameters that can be determined from experimental fitting: $w_1$is the

remaining mass fraction in the limit $s_V \to 0$, w_0 is the remaining fraction in the limit of large s_V, and β is the proposed proportionality constant. Similar exponential-decay relationships between SAR and diameter shrinkage have been proposed,[7] with a clear tendency to reach a terminal value close to $SAR \sim 100$. Interestingly, the first term in the Taylor series of w_f, centered at $s_V = 0$ and assuming no degassing limit ($w_0 = 0$), is equal to $w_f \approx 1 - \beta s_V$, stating that w_f reduces linearly with increasing s_V. One arrives to the same conclusion ($w_f \propto -s_V$) if the time dependencies in Eq. (S17) are dropped. By equating Eq. (S17) to $1 - \beta s_V$, an effective value for the constant β can be determined:

$$\beta_{\text{eff}} = \frac{j_{m,\text{eff}}\tau_{p,\text{eff}}}{\rho_i}. \tag{S21}$$

Thus, β_{eff} has length units and $j_{m,\text{eff}}$, $\tau_{p,\text{eff}}$ can be treated order of magnitude estimates of the complex mass loss process due to gas evolution. The above derivation has the shortcoming of being a purely geometrical argument for the dependence of w_f, which disregards thermodynamics of the heating process (ramp, dwell time, final temperature) and atmosphere conditions (e.g. vacuum or nitrogen flow). However, given the strong evidence that surface area is the prime contributor to feature shrinkage,[7] Eq. (S20) remains a fair estimate. In essence, this approximation serves as a simple route to analyze the shrinkage solely terms of input geometry (s_V).

a. Calculation of w_f from experimental data

To compare the derived model for w_f to the available experimental data from the literature, we note that in view of Eq. (S16), w_f may be written as:

$$w_f = \frac{\rho_f V_f}{\rho_i V_i} = \left(\frac{\rho_f}{\rho_i}\right)\frac{d_f^2 L_f}{d_i^2 L_i} \tag{S22}$$

with the subscript f denoting dimensions after pyrolysis. In terms of percent reduction, we recall that $d_{\%} = 1 - d_f/d_i$, $\delta_{\%} = (L_f - L_i)/Li$, and thus we may write alternatively:

$$w_f = \left(\frac{\rho_f}{\rho_i}\right)(1 \pm d_{\%})^2(1 \pm \delta_{\%}). \tag{S23}$$

In Fig. 5(b) in the main manuscript, w_f has been plotted by gathering d_i, d_f, L_i and L_f from several sources in the literature.

4. Pyrolysis protocol

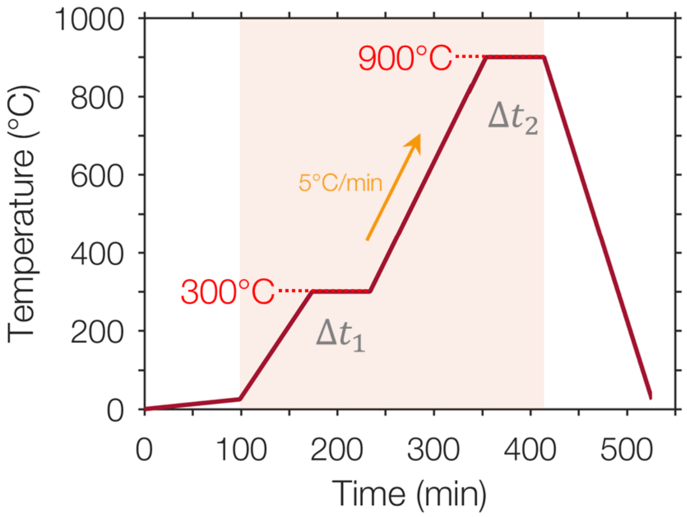

Fig. S3. Pyrolysis protocol used to fabricate the suspended carbon nanowires. The colored region in the plot indicates the parts where heat control was applied in the furnace.

5. References

1. Eschenbaum, C. Zwei-Photonen-Lithografie zur Herstellung optofluidischer Systeme. (2016).
2. Wang, I., Bouriau, M., Baldeck, P. L., Martineau, C. & Andraud, C. Three-dimensional microfabrication by two-photon-initiated polymerization with a low-cost microlaser. *Opt. Lett.* **27,** 1348–1350 (2002).
3. Serbin, J. *et al.* Femtosecond laser-induced two-photon polymerization of inorganic–organic hybrid materials for applications in photonics. *Opt. Lett.* **28,** 301 (2003).
4. Shukla, S. *et al.* Subwavelength direct laser patterning of conductive gold nanostructures by simultaneous photopolymerization and photoreduction. *ACS Nano* **5,** 1947–1957 (2011).
5. Sun, H. B., Takada, K., Kim, M. S., Lee, K. S. & Kawata, S. Scaling laws of voxels in two-photon photopolymerization nanofabrication. *Appl. Phys. Lett.* **83,** 1104–1106 (2003).
6. Gibson, I., Rosen, D. & Stucker, B. *Additive manufacturing technologies: 3D printing,*

rapid prototyping, and direct digital manufacturing, second edition. *Additive Manufacturing Technologies: 3D Printing, Rapid Prototyping, and Direct Digital Manufacturing, Second Edition* (Springer New York, 2015). doi:10.1007/978-1-4939-2113-3

7. Natu, R., Islam, M., Gilmore, J. & Martinez-Duarte, R. Shrinkage of SU-8 microstructures during carbonization. *J. Anal. Appl. Pyrolysis* **131,** 17–27 (2018).
8. Lim, Y., Heo, J., Madou, M. & Shin, H. Monolithic carbon structures including suspended single nanowires and nanomeshes as a sensor platform. *Nanoscale Res. Lett.* **8,** 1–9 (2013).
9. Canton, G., Do, T., Kulinsky, L. & Madou, M. Improved conductivity of suspended carbon fibers through integration of C-MEMS and Electro-Mechanical Spinning technologies. *Carbon N. Y.* **71,** 338–342 (2014).
10. Amato, L. *et al.* Dense high-aspect ratio 3D carbon pillars on interdigitated microelectrode arrays. *Carbon N. Y.* **94,** 792–803 (2015).
